# Supplementary material for: A multimodal neuroimaging study of brain abnormalities and clinical correlates in post treatment Lyme disease
Source: PLoS One. 2022 Oct 26;17(10):e0271425. doi: 10.1371/journal.pone.0271425 (PMC9604010; doi:10.1371/journal.pone.0271425)
Supplement: S1 Table — Brain regions are based on Talairach coordinates. Bold indicates activations that overlapped with a prior study in young, healthy adults using the same fMRI task by Marvel & Desmond, 2012. All regions met threshold criteria for p < .001, uncorrected. (DOCX) [file pone.0271425.s001.docx]

**S1 Table**

**FMRI task BOLD activations within healthy controls**

| **Cluster size** | ***T*-value** | ***x*, *y*, *z* (MNI)** | **Brain region (BA)** |  |  |
| --- | --- | --- | --- | --- | --- |
| **(*n*=18)**   \| 20 \| 4.26 \| -44, 44, 14 \| L Middle frontal gyrus (BA 10) \| \| --- \| --- \| --- \| --- \| \| 193 \| 7.16 \| **40, 36, 30** \| R Superior frontal gyrus (BA 9) \| \| 201 \| 4.71 \| **-32, 18, 2** \| L Anterior insula (BA 13) \| \| 680 \| 8.05 \| **36, 14, 2** \| R Anterior insula (BA 13) \| \| 2451 \| 6.44 \| **12, 14, 44** \| R Middle frontal gyrus (BA 32) \| \| 1044 \| 5.38 \| **-48, 4, 22** \| L Precentral gyrus (BA 6) \| \| 18 \| 3.83 \| **10, 2, 20** \| R Caudate \| \| 31 \| 5.09 \| -8, -12, 76 \| L Superior frontal gyrus (BA 6) \| \| 2598 \| 6.11 \| **6, -24, 12** \| Thalamus \| \| 4558 \| 7.53 \| **44, -38, 42** \| R Inferior parietal lobule (BA 44) \| \| 77 \| 5.56 \| -50, -50, -12 \| L Inferior temporal gyrus (BA 20) \| \| 347 \| 4.79 \| **-32, -62, -28** \| L Cerebellar crus I \| \| 1315 \| 5.68 \| **36, -68, -22** \| R Cerebellar crus I \| \| 114 \| 4.96 \| 28, -68, 6 \| R Posterior cingulate (BA 30) \| \| 59 \| 4.18 \| -22, -76, -50 \| L Cerebellar crus II \|  \| **Brain regions are based on Talairach coordinates.** Bold indicates activations that overlapped with a prior study in young, healthy adults using the same fMRI task by Marvel & Desmond, 2012. All regions met threshold criteria for *p* < .001, uncorrected. \|  \|  \| \| --- \| --- \| --- \| | | | |  |  |
