# Supplementary material for: A multimodal neuroimaging study of brain abnormalities and clinical correlates in post treatment Lyme disease
Source: PLoS One. 2022 Oct 26;17(10):e0271425. doi: 10.1371/journal.pone.0271425 (PMC9604010; doi:10.1371/journal.pone.0271425)
Supplement: S1 File — Table 1 in S1 File shows descriptive statistics for fMRI, DTI, and clinical variables. Table 2 in S1 File shows descriptive statistics for gray matter fMRI data and scores on the Beck Depression Inventory (BDI). In both tables, data are shown as: mean (standard deviation), 95% confidence interval [lower limit, upper limit]. Shapiro-Wilk tests were used for tests of normality. Significant values of the Shapiro-Wilk tests are denoted in bold, p ≤ .05, two-tailed. Table 3 in S1 File reports correlations between the fMRI ROIs and their respective DTI ROIs. Pearson’s tests were used for bivariate correlations involving BA 9 values because they have a normal distribution. A Spearman’s test was used for bivariate correlations involving BA 8 values because they had a non-normal distribution. Table 4 in S1 File reports correlations between fMRI and DTI ROIs and fMRI Task Accuracy, RT, Symptoms, and BDI. Spearman’s tests were used for bivariate correlations involving Task Accuracy, RT, Cognitive Symptoms, and BDI because they had a non-normal distribution. Pearson’s tests were used for bivariate correlations involving Total Clinical Symptoms and Neurological Symptoms because they had a normal distribution. (DOCX) [file pone.0271425.s003.docx]

**Table 1: FMRI, DTI, and Clinical Variable Descriptive Statistics**

|  | PTLD | Controls |
| --- | --- | --- |
| fMRI ROI:  BA 8  BA 9 anterior  BA 9 posterior | .009 (.012), 95% [.002, .016], W = .94, p = .52  .009 (.017), 95% [-.002, .021], W = .96, p = .73  .017 (.020), 95% [.004, .031], W = .95, p = .66 | -.007 (.012), 95% [-.013, -.001], W = .94, p = .34  -.010 (.016), 95% [-.018, -.002], W = .95, p = .45  -.006 (.016), 95% [-.013, .002], W = .93, p = .20 |
| Axial diffusivity ROI:  BA 8  BA 9 anterior  BA 9 posterior | .001 (.00008), 95% [.001, .001], W = .97, p = .93  .001 (.00006), 95% [.001, .001], W = .94, p = .43  .001 (.00002), 95% [.001, .001], W = .95, p = .63 | .001 (.00008), 95% [.001, .001], W = .83, **p = .019**  .001 (.00008), 95% [.001, .001], W = .98, p = .99  .001 (.0001), 95% [.001, .001], W = .89, p = .11 |
| PLQS:  Total  Neurologic  Cognitive | 8.17 (4.39), 95% [5.38, 10.95], W = .94, p = .48  4.50 (2.023) 95% [3.21, 5.79], W = .90, p = .16  2.00 (1.35), 95% [1.14, 2.86], W = .71, **p = .001** | ---------- |
| FMRI task  *Accuracy:*  Control condition  1 stimulus  2 stimuli  Forward condition  1 stimulus  2 stimuli  *Response time:*  Control condition  1 stimulus  2 stimuli  Forward condition  1 stimulus  2 stimuli | 97.8 (2.00), 95% [96.4, 99.1], W = .79, **p = .008**  97.2 (2.60), 95% [95.4, 98.9], W = .72, **p = .001**  94.9 (10.0), 95% [88.2, 101.6], W = .55, **p < .001**  89.8, (12.0), 95% [88.2, 101], W = .70, **p = .001**  1102 (326), 95% [883, 1321], W = .87, p = .075  1226 (417), 95% [946, 1506], W = .75, **p = .002**  1221 (455), 95% [946, 1527], W = .84, **p = .034**  1800 (632), 95% 1375, 2225), W = .83, **p = .025** | 96.9 (5.02), 95% [94.4, 99.4], W = .66, **p < .001**  95.7 (6.35), 95% [92.5, 98.8], W = .71, **p < .001**  96.0 (3.69), 95% [94.2, 97.8], W = .84, **p = .007**  91.3 (4.75), 95% [89.0, 93.7], W = .94, p = .26  922 (184), 95% [831, 1014], W = .98, p = .94  1011 (198), 95% [913, 1110], W = .89, **p = .039**  996 (217), 95% [888, 1104], W = .98, p= .97  1373 (384), 95% [1182, 1564], W = .96, p = .64 |

Data are shown as: mean (standard deviation), 95% confidence interval [lower limit, upper limit]. Shapiro-Wilk tests were used for tests of normality. Significant values of the Shapiro-Wilk tests are denoted in bold, p < .05, two-tailed.

**Table 2: Gray Matter fMRI and Beck Depression Inventory (BDI) Descriptive Statistics**

|  | PTLD |
| --- | --- |
| fMRI ROI:  Left Medial Frontal Gyrus (BA9)  Left Precentral Gyrus (BA6)  Right Thalamus | .019 (.041), 95% [-.008, .047], W = .97, p = .916  .009 (.021), 95% [-.005, .023], W = .93, p = .452  .014 (.028), 95% [-.005, .032], W = .88, p = .090 |
| BDI Total Score | 18.42 (12.05), 95% [10.76, 26.07], W = .79, **p = .008** |

Data are shown as: mean (standard deviation), 95% confidence interval [lower limit, upper limit]. Shapiro-Wilk tests were used for tests of normality. Significant values of the Shapiro-Wilk tests are denoted in bold, p < .05, two-tailed.

**Table 3: Correlations Between fMRI ROIs and Respective DTI ROIs**

| *ROIs* | *DTI BA 8* | *DTI BA 9 anterior* | *DTI BA 9 posterior* |
| --- | --- | --- | --- |
| fMRI BA 8 | r(11) = .44, p = .18 |  |  |
| fMRI BA 9 anterior |  | **r(11) = -.55, p = .077** |  |
| fMRI BA 9 posterior |  |  | r(11) = .11, p = .76 |

Pearson’s tests were used for bivariate correlations involving BA 9 values because they have a normal distribution. A Spearman’s test was used for bivariate correlations involving BA 8 values because they had a non-normal distribution.

**Table 4: Correlations Between fMRI and DTI ROIs and fMRI Task Accuracy, RT, Symptoms, and BDI**

| *Region of Interest (ROI)* | *Task Accuracy* | *Task Reaction Time (RT)* | *Total Clinical Symptoms* | *Neurological Symptoms* | *Cognitive Symptoms* | *Beck Depression Index Total Score (BDI)* |
| --- | --- | --- | --- | --- | --- | --- |
| fMRI BA 8 | r(10) = -.54  p = .10 | r(10) = .18  p = .60 | r(11) = .19  p = .57 | r(11) = .22  p = .52 | r(11) = .26  p = .45 | r(11) = -0.352  p = 0.289 |
| fMRI BA 9 Anterior | r(10) = -.42  p = .22 | r(10) = -.055  p = .88 | r(11) = -.059  p = .86 | r(11) = -.11  p = .75 | r(11) = .000  p = 1.00 | r(11) = -0.374  p = 0.257 |
| fMRI BA 9 Posterior | r(10) = -.24  p = .51 | r(10) =.07  p = .83 | r(11) = -.035  p = .92 | r(11) =.032  p = .93 | r(11) = .085  p = .80 | r(11) = -0.584  p = 0.059 |
| fMRI Left Medial Frontal Gyrus (BA9) | r(10) = -.30  p = .40 | r(10) = .07  p = .86 | r(11) = -.12  p = .72 | r(11) = -.097  p = .78 | r(11) = -.085  p = .80 | r(11) = -0.584  p = 0.059 |
| fMRI Left Precentral Gyrus (BA6) | r(10) = -.20  p = .58 | r(10) = .02  p = .96 | r(11) = -.006  p = .99 | r(11) = .012  p = .97 | r(11) = -.021  p = .95 | **r(11) = -0.767**  **p = 0.006** |
| fMRI Right Thalamus | r(10) = -.31  p = .39 | r(10) =.15  p = .68 | r(11) = .097  p = .78 | r(11) = -.01  p = .978 | r(11) = .23  p = .49 | r(11) = -0.151  p = 0.658 |
| DTI BA 8 | r(11) = -.30  p = .37 | r(11) = -.027  p = .94 | r(12) = .10  p = .75 | r(12) = -.086  p = .79 | r(12) = -.33  p = .29 | r(11) = -0.207  p = 0.519 |
| DTI BA 9 Anterior | r(11) = .41  p = .22 | r(11) = .064  p = .85 | **r(12) = -.76**  **p = .004** | **r(12) = -.78**  **p = .003** | **r(12) = -.81**  **p = .001** | r(11) = -0.316  p = 0.317 |
| DTI BA 9 Posterior | r(11) = .25  p = .45 | **r(11) = -.63**  **p = .039** | r(12) = -.20  p = .54 | r(12) =.058  p = .86 | r(12) = -.17  p = .61 | r(11) = 0.056  p = 0.862 |
| Beck Depression Index Total Score (BDI) | r(11) = .15  p = .66 | r(11) = -.15  p = .66 | **r(12) = .59**  **p = .044** | **r(12) = .64**  **p = .024** | r(12) = .37  p = .24 | ------- |

Spearman’s tests were used for bivariate correlations involving Task Accuracy, RT, Cognitive Symptoms, and BDI because they had a non-normal distribution. Pearson’s tests were used for bivariate correlations involving Total Clinical Symptoms and Neurological Symptoms because they had a normal distribution.
